# Exploring the metabolic network of the epidemic pathogen *Burkholderia cenocepacia *J2315 via genome-scale reconstruction

**DOI:** 10.1186/1752-0509-5-83

**Published:** 2011-05-25

**Authors:** Kechi Fang, Hansheng Zhao, Changyue Sun, Carolyn M C Lam, Suhua Chang, Kunlin Zhang, Gurudutta Panda, Miguel Godinho, Vítor A P Martins dos Santos, Jing Wang

**Affiliations:** 1 Key Laboratory of Mental Health, Institute of Psychology, Chinese Academy of Sciences, Beijing 100101, China; 2 College of Biological Sciences, China Agricultural University, Beijing 100193, China; 3 Systems and Synthetic Biology Group, Helmholtz Center for Infection Research (HZI), Inhoffenstrasse 7, 38124 Braunschweig, Germany; 4 Graduate University of Chinese Academy of Sciences, Beijing 100101, China; 5 Lifewizz Lda, Rua Pero de Alenquer 123 7 E, 4150-616 Porto, Portugal; 6 Systems and Synthetic Biology, Wageningen University, Dreijenplein 10, 6703 HB Wageningen, The Netherlands

## Abstract

**Background:**

*Burkholderia cenocepacia *is a threatening nosocomial epidemic pathogen in patients with cystic fibrosis (CF) or a compromised immune system. Its high level of antibiotic resistance is an increasing concern in treatments against its infection. Strain *B. cenocepacia *J2315 is the most infectious isolate from CF patients. There is a strong demand to reconstruct a genome-scale metabolic network of *B. cenocepacia *J2315 to systematically analyze its metabolic capabilities and its virulence traits, and to search for potential clinical therapy targets.

**Results:**

We reconstructed the genome-scale metabolic network of *B. cenocepacia *J2315. An iterative reconstruction process led to the establishment of a robust model, *i*KF1028, which accounts for 1,028 genes, 859 internal reactions, and 834 metabolites. The model *i*KF1028 captures important metabolic capabilities of *B. cenocepacia *J2315 with a particular focus on the biosyntheses of key metabolic virulence factors to assist in understanding the mechanism of disease infection and identifying potential drug targets. The model was tested through BIOLOG assays. Based on the model, the genome annotation of *B. cenocepacia *J2315 was refined and 24 genes were properly re-annotated. Gene and enzyme essentiality were analyzed to provide further insights into the genome function and architecture. A total of 45 essential enzymes were identified as potential therapeutic targets.

**Conclusions:**

As the first genome-scale metabolic network of *B. cenocepacia *J2315, *i*KF1028 allows a systematic study of the metabolic properties of *B. cenocepacia *and its key metabolic virulence factors affecting the CF community. The model can be used as a discovery tool to design novel drugs against diseases caused by this notorious pathogen.

## Background

*Burkholderia cenocepacia *is a Gram-negative opportunistic pathogen and formerly Genomovar III of *Burkholderia cepacia *complex (Bcc). The Bcc comprises at least 17 taxonomically related species [1-3], which have developed diverse niches from the natural environment [4] and humans as they have emerged as pathogens in patients with cystic fibrosis (CF), chronic granulomatous disease, and in immunocompromised individuals [5]. *B. cenocepacia *is the dominant Bcc species in patients with CF, accounting for between 50% and 80% of the infection cases [5]. It also causes many instances of non-CF clinical infections, such as for cancer patients [6,7]. As a representative isolate for the spread of an epidemic CF strain, *B. cenocepacia *J2315 belongs to a clonal lineage known as ET12, which is of increased transmissibility and dominates fatal infections among CF patients in the United Kingdom and Canada [8-12]. *B. cenocepacia *J2315 is notorious for its high resistance to the majority of clinically useful antimicrobial agents [6,13], including antimicrobial peptides [14,15]. Yet the mechanisms of host infection and drug resistance remain mostly unknown.

The genome of *B. cenocepacia *J2315 has been sequenced and recently annotated [13]. It is one of the largest Gram-negative genomes consisting of three circular chromosomes with 3.8, 3.2 and 0.8 million base pairs (Mb) respectively and a plasmid. Its complex genome encodes a broad range of metabolic capabilities, and numerous virulence and drug resistance functions that allow it to survive under a variety of conditions and invade immunocompromised individuals. It is vital to develop a systems-level metabolic model for this opportunistic human pathogen to explore and gain insights into its versatile metabolic capability and disease-causing mechanism, and eventually aid in finding potential clinical therapeutic targets. The genome-scale metabolic reconstruction enables integration of genomic information with metabolic activities observed in phenotypic experiments and other "omics" measurements to elicit hidden biological knowledge that would have been otherwise difficult to obtain.

In this study, we presented the manually curated genome-scale metabolic network of *B. cenocepacia *J2315, named as *i*KF1028, which accounts for the major metabolic pathways for the synthesis of each component of biomass and for the degradation of common biologically important carbon sources. Syntheses pathways for key virulence factors highly associated with metabolism were particularly emphasized and reconstructed. The *in silico *model was validated by performing BIOLOG substrate utilization assays, which can test the ability of a microorganism to oxidize various substrates simultaneously [16]. Model-driven analysis and discoveries, including refinement of gene annotation, and gene and enzyme essentiality, were carried out to define the architecture of the genome-wide metabolic and transport network and assist the identification of potential drug targets. Model *i*KF1028 provides researchers a framework to explore and understand the global metabolism of *B. cenocepacia *J2315 and its key metabolic virulence factors affecting CF patients upon infection. It allows a broad spectrum of basic and practical applications, especially the application for drug design which may open new doors for anti-infection strategies.

## Results and discussion

### Characteristics of the genome-scale metabolic network of *B. cenocepacia *J2315

**Figure 1:**
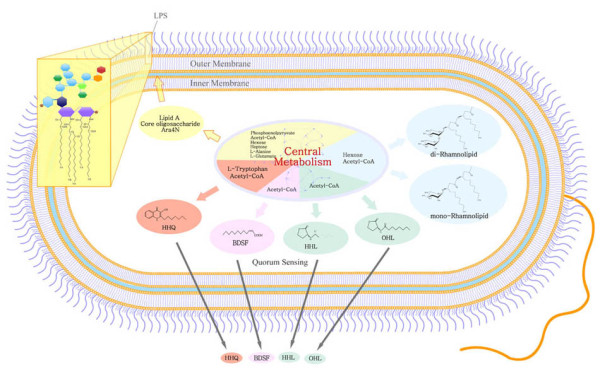
**Schematic representation of the metabolic network in *B. cenocepacia *J2315, referred as model *i*KF1028**.

**Figure 2:**
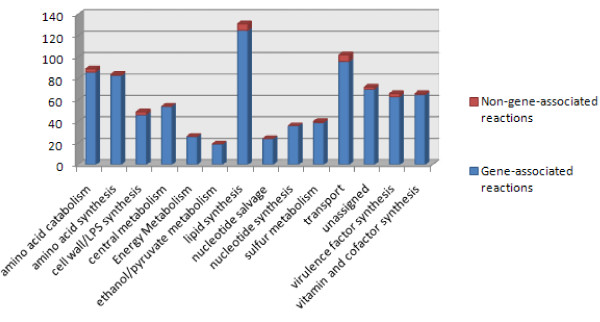
**Metabolic pathways included in *i*KF1028 and the distribution of gene-associated and non-gene-associated reactions for each pathway**.

The genome-scale reconstructed metabolic model of *B. cenocepacia *J2315, referred by the conventional naming rules [17] as *i*KF1028, consists of 859 internal reactions (including transport) and 834 metabolites. The reconstruction accounts for 1,028 genes, covering 14.4% of the 7,116 protein coding genes identified from whole genome sequencing (http://www.ncbi.nlm.nih.gov/genome?term=burkholderia%20cenocepacia%20J2315). The model *i*KF1028 includes all major pathways required for cell growth and the degradation of common biologically important carbon sources of *B. cenocepacia*. Apart from these central metabolic pathways, model *i*KF1028 also includes pathways associated with key metabolic virulence factors, which provides insights into how the system-level metabolic properties affect pathogenicity. For an overview, the properties of the J2315 genome and the reconstructed model *i*KF1028 were summarized in Table 1. Genome-scale metabolic models have been successfully used to study many pathogenic bacteria, including *Staphylococcus aureus *[18-20], *Acinetobacter baumannii *[21], *Mycobacterium tuberculosis *[22], *Salmonella typhimurium *[23], and *Pseudomonas aeruginosa *[24]. A basic comparison between the model *i*KF1028 and the above five recently published metabolic reconstructions is also illustrated in Table 1. Schematic representation of the metabolic network of *B. cenocepacia *J2315 with key metabolic virulence factors is shown in Figure 1. Figure 2 enumerates the metabolic pathways included in *i*KF1028 and, for each pathway, the number of reactions with assigned and non-assigned genes. The high ratio of gene-associated reactions shows that the reconstructed metabolic model of *B. cenocepacia *J2315 is reliable. [Additional file 1 for *i*KF1028 and Additional file 2 is in SBML format]

**Table 1:** Comparison of properties of reconstructed metabolic network for selected pathogens

Model	N.A.*	AbyMBEL891	*i*NJ661	*i*RR1083	*i*MO1056	*i*KF1028
Genome size	2.8 Mb	3.93 Mb	4.4 Mb	4.8 Mb	6.3 Mb	8.1 Mb
Included genes	758	650	661	1,083	1,056	1,028
Total reactions	1,497	891	939	1,087	883	859
Gene-associated reactions (% of total reactions)	1,278	713 (80%)	723 (77%)	1,018 (93.7%)	839 (95%)	832 (96.9%)
Non-gene-associated reactions	219	46	216	69	44	27
Transport reactions	146	130	93	230	133	102
Metabolites	1,431	778	828	744	760	834

Properties of metabolic reconstruction of *B. cenocepacia* J2315 (iKF1028) were compared with other  published metabolic reconstructions of pathogenic microbes, *S. aureus* N315 (2009) [18] (which is the  improvement of the N315 reconstruction by Becker and Palsson 2005 [19], and Heinermann *et al*. 2005  [20]), *A. baumannii* AYE (AbyMBEL891) (2010) [21], *M. tuberculosis* H37Rv (iNJ661) (2007) [22], *S.  typhimurium* LT2 (iRR1083) (2009) [23], and *P. aeruginosa* PAO1 (iMO1056) (2008) [24]. *: Not  available for the reconstruction of *S. aureus* N315.

### Metabolic virulence factors in model *i*KF1028

The success of *B. cenocepacia *as a pathogen originates from the ability of its large genome to encode numerous virulence mechanisms [13], including quorum sensing (QS) [25-30], siderophores-based iron uptake systems [31-33], cable pili and adhesion [34-36], motility [37,38], hemolysin [39], ZmpA and ZmpB proteases [40-42], phospholipases [43], secretion systems [44-46], lipopolysaccharides (LPS) [15,47-49], and extracellular capsule [50]. Syntheses of the key metabolic virulence factors of these virulent mechanisms, namely QS, LPS and rhamnolipids, were incorporated and analyzed in *i*KF1028. Table 2 lists the virulence-associated pathways and the required proteins and precursors for syntheses of virulence factors in each pathway.

**Table 2:** Virulence factors incorporated in the metabolic network reconstruction of *B. cenocepacia *J2315

Virulence factors	Proteins involved	Major metabolic precursors
*Lipopolysaccharide components*		
Lipid A	LpxA, LpxB, LpxC, LpxD, LpxH, LpxK, KdsA, KdsB, KdsC, KdtA, KdoO, HtrB	O_2,_ UDP-N-acetyl-D-glucosamine, (*R*)-3-Hydroxytetradecanoyl-ACP, (*R*)-3-Hydroxyhexadecanoyl-ACP, Myristoyl-ACP, D-arabinose 5-phosphate, Phosphoenolpyruvate,
Core oligosaccharide	GmhA, RfaE, GmhB, HldD, RmlD, WbiI, WaaC, WaaF, WabP, WabR, WabO, WabS, WaaL	Sedoheptulose 7-phosphate, UDP-glucose, UDP-N-acetyl-D-glucosamine, dTDP-4-dehydro-6-deoxy-L-mannose, L-Alanine
Ara4N modification	ArnA1, ArnA2, ArnB, ArnC, ArnT	L-Glutamate, 10-Formyltetrahydrofolate, UDPglucuronate
*Quorum sensing*		
AHLs	CepI, CciI	S-adenosyl-L-methionine, Octanoyl-ACP, Hexanoyl-ACP
HHQ	KynA, KynB, KynU, PqsA, PqsB, PqsC, PqsD	L-Tryptophan, 3-oxodecanoyl-ACP
BDSF	RpfF, FadA, FadB, FadH	(*S*)-Hydroxydecanoyl-CoA
*Rhamnolipids*	RhlA, RhlB, RhlC, PhaC	dTDP-4-dehydro-6-deoxy-L-mannose, (*R*)-3-Hydroxydecanoyl-ACP

Ara4N, 4-amino-4-deoxy-arabinose; AHLs, *N*-acylhomoserine lactones; HHQ, 2-heptyl-4-quinolone; BDSF, *cis*-2-dodecenoic acid

**Figure 3:**
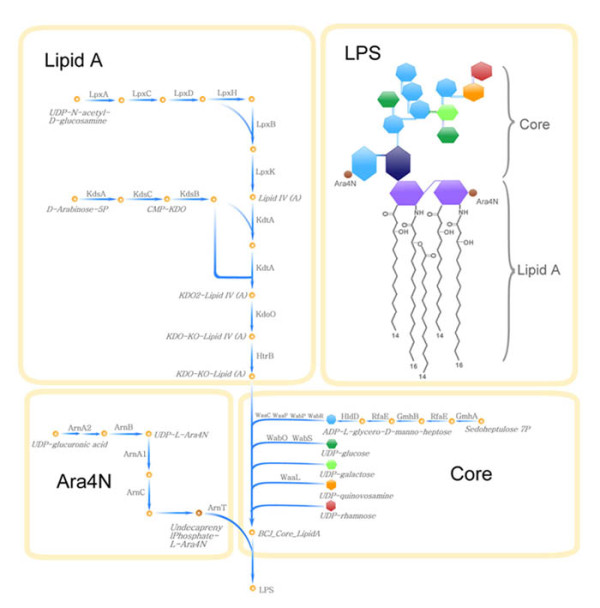
**Specific structure of Lipopolysaccharide (LPS) in *B. cenocepacia *J2315 and the synthesis pathways of LPS as well as the proteins involved**. The lipid A portion of LPS is composed of two linked glucosamine residues (purple hexagon) with fatty acid side chains (wavy lines), (*R*)-3-hydroxyhexadecanoic (C16:0 (3-OH)) in an amide linkage and (*R*)-3-hydroxytetradecanoic (C14:0 (3-OH)) acid and tetradecanoic acid (C14:0) in an ester linkage. There are 4-amino-4-deoxyarabinose (Ara4N, brown sphere) moieties attached to the phosphate residues in the lipid A backbone. The inner core oligosaccharide contains unusual KDO-KO-Ara4N residue linked to the lipid A (KDO: 3-deoxy-D-*manno*-octulosonic acid, dark blue hexagon; KO: D-*glycero*-D-*talo*-octulosonic acid, light blue hexagon). Various polysaccharides comprise the outer core oligosaccharide (L-glycero-D-*manno*-heptose, blue heptagon; glucose, dark green hexagon; galactose, light green hexagon; quinovosamine, orange hexagon; rhamnose, red hexagon). J2315 cannot make complete LPS O-antigen, owing to an insertion element in BCAL3125 [47].

The LPS produced by *B. cenocepacia *J2315 has an important role in both disease aetiology and antibiotic resistance [51,52]. LPS usually consists of three components: lipid A, core oligosaccharide, and O antigen. Although there were some studies on characterizing the features of LPS in *B. cenocepacia*, all these studies focused on a certain part/component of LPS. So far, there is no systematic elucidation of the LPS structure and composition specifically for *B. cenocepacia *strain J2315, nor any global analysis on its biosynthesis process of the LPS. In this study, we depicted the detailed features of the complete LPS structure in *B. cenocepacia *J2315 by integrating all available reports on LPS. We also reconstructed the LPS-synthesis pathways supplemented with all necessary proteins involved and major metabolic precursors, as illustrated in Figure 3. According to our study, in J2315, each of the three components has a very unique feature. The lipid A portion is modified by an additional Ara4N residue [49,53], which had been shown to reduce the binding of cationic antibiotics and was proposed as a potential drug target [54]. The inner core oligosaccharide contains an unusual KDO-KO-Ara4N residue instead of the typical KDO-KDO residue [15,51,55]. The outer core comprises various polysaccharides including L-glycero-D-manno-heptose, glucose, galactose, quinovosamine, and rhamnose [51]. The O-antigen portion of LPS in J2315 was interrupted by an insertion element in BCAL3125 [47,56]. These differences might indicate the reason why strain J2315 is of remarkably distinct activity.

*B. cenocepacia *strains possess multiple quorum sensing systems, which regulate the expression of versatile virulence determinants, such as biofilm formation and motility. Strain J2315 owns the ability to synthesize and recognize three types of chemical signals used for cell-to-cell communication: *N*-acylhomoserine lactones (AHLs), 4-quinolones (4Qs), and the DSF-like molecule *cis*-2-dodecenoic acid (BDSF). Two AHLs-based QS systems have been found in J2315, namely CciIR and CepIR [25,57], which can both produce *N*-hexanoyl-L-homoserine lactone (C6-HSL) and *N*-octanoyl-L-homoserine lactone (C8-HSL) signals using acyl side chain (Hexanoyl-ACP and Octanoyl-ACP, respectively) and *S*-adenosyl-methionine (SAM) as precursors [58]. The CepIR system is conserved in all species of the Bcc. The CciIR system is encoded within a pathogenicity island, designated as the *B. cenocepacia *island (cci), which was the first time that cell-signalling genes were found on a genomic island [59]. The 4Qs-based signal, the 2-heptyl-4-quinolone (HHQ), is produced by *B. cenocepacia *strains [26]. HHQ is the precursor of 2-heptyl-3-hydroxy-4-quinolone (PQS) [60] and its synthesis requires four proteins: PqsA, PqsB, PqsC, and PqsD. It had been reported that the exported HHQ from *B. cenocepacia *can be recognized by *Pseudomonas aeruginosa *within which HHQ is converted into PQS which is one of the QS signals for *P. aeruginosa *[26], highlighting the possibility of inter-species communication during the CF co-infection caused by *P. aeruginosa *and *B. cenocepacia*. BDSF is a newly discovered signal molecule produced by *B. cenocepacia *[28]. The synthesis of BDSF requires the gene BCAM0581 [29].

The synthesis pathway of rhamnolipids was also reconstructed in *i*KF1028. Although there has not been any report demonstrating that *B. cenocepacia *can produce rhamnolipid, Dubeau *et al *demonstrated that *Burkholderia thailandensis *has the orthologs of rhlA, rhlB, and rhlC, which are responsible for the biosynthesis of rhamnolipids in *P. aeruginosa *[61]. By protein similarity search against the UniProt database, proteins coded by genes BCAM2340, BCAM2338, and BCAM2336 in *B. cenocepacia *J2315 were identified as highly similar in sequence to rhlA, rhlB, and rhlC in both *B. thailandensis *(with BLAST E value of 1E-121, 1E-173, and 1E-108, respectively) and *P. aeruginosa *(with BLAST E value of 3E-60, 7E-98, and 1E-67, respectively). This facilitates us to hypothesize that *B. cenocepacia *can potentially generate rhamnolipids. Further experimental investigations are needed.

### Model validation and gap-filling using phenotype data

BIOLOG substrates utilization assays for *B. cenocepacia *J2315 were performed in triplicates in order to validate and refine the model. *In silico *growth on various substrates was simulated by setting each of them as sole carbon source and its uptake rate to 10 mmol/g_cell_/h under aerobic conditions based on M9 minimal medium. The simulation was performed on the ToBiN platform by flux balance analysis, as described in Methods. Of the 95 carbon sources tested, 40 could be directly compared with the *in silico *model of *B. cenocepacia *J2315, *i*KF1028. Preliminary disagreement between BIOLOG assays and *in silico *predictions were probably due to metabolic gaps, improper gene annotations and unacquainted transporters. These discrepancies were checked through gap analysis and literature mining. After continuous gap-filling and network refinement, the overall prediction accuracy was improved to 87.5%, a value that supported *i*KF1028 as being a proper reconstruction of the *B. cenocepacia *J2315 core metabolism (comparison results are showed in Table 3).

**Table 3:** Comparison with the BIOLOG substrates utilization assays

Class	Carbon source	BIOLOG results	*In silico *prediction	Agreement
Carbohydrates	*N*-Acetyl-D-glucosamine	No Growth	No Growth	yes
	D-Galactose	Growth	Growth	yes
	α-D-Glucose	Growth	Growth	yes
	*m*-Inositol	No Growth	No Growth	yes
	Sucrose	Growth	Growth	yes
	D-Trehalose	Growth	Growth	yes

Carboxylic acids	Acetic acid	Growth	Growth	yes
	*cis*-Aconitic acid	Growth	Growth	yes
	Citric acid	Growth	Growth	yes
	D-Gluconic acid	Growth	Growth	yes
	β-Hydroxybutyric acid	Growth	Growth	yes
	α-Ketoglutaric acid	Growth	Growth	yes
	D,L-Lactic acid	Growth	Growth	yes
	Malonic acid	Growth	Growth	yes
	Propionic acid	No Growth	No Growth	yes
	Quinic acid	Growth	Growth	yes
	D-Saccharic acid	Growth	Growth	yes
	Succinic acid	Growth	Growth	yes

Amino acids	L-Alanine	Growth	Growth	yes
	L-Asparagine	Growth	Growth	yes
	L-Aspartic acid	No Growth	Growth	no
	L-Glutamic acid	Growth	Growth	yes
	L-Histidine	Growth	Growth	yes
	Hydroxy-L-proline	Growth	Growth	yes
	L-Leucine	No Growth	Growth	no
	L-Ornithine	No Growth	Growth	no
	L-Phenylalanine	Growth	Growth	yes
	L-Proline	Growth	Growth	yes
	L-Pyroglutamic Acid	Growth	Growth	yes
	L-Serine	Growth	Growth	yes
	L-Threonine	No Growth	Growth	no
	D,L-Carnitine	No Growth	No Growth	yes
	γ-Aminobutyric acid	Growth	Growth	yes

Miscellaneous	Succinamic acid	Growth	Growth	yes
	Uridine	No Growth	No Growth	yes
	Thymidine	No Growth	No Growth	yes
	Putrescine	No Growth	No Growth	yes
	2,3-Butanediol	No Growth	No Growth	yes
	Glycerol	No Growth	Growth	no
	D-Glucose-6-Phosphate	Growth	Growth	yes

Of the remaining 55 carbon sources tested, 14 were indirectly compared with the model due to the missing knowledge of whether the transport mechanisms of these compounds exist in J2315 or not. Initially, all those 14 carbon sources showed a no-growth phenotype both *in silico *and in the BIOLOG assays. Then we made the assumption that each of these carbon sources could be transported into the cell (*i.e. *to function as intracellular compounds) and re-tested whether the *in silico *model can grow on each of them. The results showed that 11 of the 14 carbon sources enabled *i*KF1028 to grow after applying the above assumption. This supports the hypothesis that J2315 lacks of transporters for all those 11 carbon sources, even though their catabolic pathways are complete. For the rest 3 carbon sources, the agreement between the *in silico *results and BIOLOG assays remained.

As the catabolism of the remaining 41 carbon sources out of 55 has not been well studied and information regarding their role in the cell could not be found in public resources, these 41 carbon sources cannot be analyzed in our model. (Complete comparison results with BIOLOG assays are supplied in the Additional file 3)

### Model-driven refinement of genome annotation

The reconstruction of metabolic network allowed the identification and refinement of improperly annotated genes of *B. cenocepacia *J2315 from the public biological databases. Careful effort was made in this work to rectify the current genome annotation based on metabolic gap analysis, BLAST searches, BIOLOG substrate utilization assays, and literature mining. The full list of refinement of genome annotation derived from the genome-scale metabolic reconstruction is shown in Table 4.

**Table 4:** Proposed annotation refinements

Gene Locus	Current Annotation (Burkholderia.com)	Proposed Reannotation	Protein name	Protein ID	Evidence
BCAL0691, BCAL2945	Putative cytidylyltransferase, D-beta-D-heptose 7-phosphate kinase	Bifunctional protein RfaE domain II and I, respectively, sugar kinase/adenylyltransferase	RfaE	-	Modelling evidence, RfaE is necessary for biosynthesis of ADP-L-glycero-D-manno-heptose, a precursor for LPS inner core biosynthesis; BLAST search of RfaE from *P. aeruginosa *gave E values of 9E-35 and 1E-75, respectively
BCAL0780	Putative multiphosphoryl transfer protein	Glucose-specific enzyme IIA component of PTS	Crr	TC-4.A.1.1.1	BIOLOG assays indicated growth on glucose; BLAST search of Crr from *E.coli *gave an E value of 1E-28 and Identities of 40%
BCAL0781	Phosphotransferase system, IIbc component	Glucose/N-acetyl glucosamine-specific IIC component	PtsG/NagE	-	Evidence from BIOLOG assays; BLAST search of PtsG, NagE from *E.coli *gave E values of 3E-107, 7E-151 and Identities of 43%, 56%, respectively
BCAL0802	Gene locus is not assigned in *Burkholderia *Genome Database and KEGG	4-diphosphocytidyl-2-C-methyl-D-erythritol kinase	IspE	EC-2.7.1.148	Modelling evidence, IspE is necessary for biosynthesis of polyprenyl-PP, a precursor for ubiquinone biosynthesis; BLAST E value of 4E-172; assigned gene locus of BCAL0802 (from 872938 to 873820) in GeneDB database
BCAL1281	Hypothetical protein	Ornithine *N*-acyltransferase	OlsB	EC-2.3.1.-	Physiological evidence from Weissenmayer *et al*. (2002); OlsB is required for the first step of ornithine-derived lipid biosynthesis; BLAST E value of 1E-29
BCAL1431, BCAL1432, BCAL1433	Putative sugar transport system	Galactose transport	MglB, MglA, MglC	TC-3.A.1.2.3	BIOLOG assays indicated growth on galactose; and BLAST E values (< 2E-23)
BCAL1933, BCAL1934	Putative formyltransferase, NAD-dependent epimerase/dehydratase family protein	UDP-Ara4N formyltransferase, UDP-4'-keto-5'-carboxypentose decarboxylase	ArnA1, ArnA2	-	Evidence from Ortega *et al*. (2006). Unlike other bacteria in which arnA is a single gene encoding a bifunctional enzyme, two distinct genes were found in J2315 (arnA1 and arnA2) and both are required for Ara4N biosynthesis
BCAL2388	Hypothetical protein	Glucose-1-phosphate adenylyltransferase	-	EC-2.7.7.27	Modelling evidence, a missing protein is required for glycogen biosynthesis; and BLAST search against UNIPROT database gave an E value of 9E-58
BCAL3280	Putative carbon-nitrogen hydrolase protein	Succinamic acid amidohydrolase	-	EC-3.5.1.3	BIOLOG assays indicated growth on succinamic acid; modelling showed a missing protein in this pathway; BLAST search against UNIPROT database gave an E value of 3E-46
BCAL3365	Putative gluconate permease	D-gluconate: H+ symporter	GntP	TC-2.A.8.1.3	BIOLOG assays indicated growth on D-Gluconic acid; modelling revealed a lack of transporter; BLAST E values of 4E-68
BCAM0469	Putative aldehyde dehydrogenase	Aldehyde dehydrogenase A, NAD-linked	AldA	EC-1.2.1.21	Modelling evidence: a gene is missing to synthesize glycolaldehyde which is required for biosynthesis of vitamin B6; BLAST E value of 2E-74
BCAM1404	Probable exported glycosyl hydrolase	Sucrose-6-phosphate hydrolase	ScrB	EC-3.2.1.26	BIOLOG assays indicated growth on sucrose; modelling showed missing protein along the pathway; gene locus identified from annotation as 93% similarity from *Staphylococcus aureus *and E value of 1E-33
BCAM2340, BCAM2338, BCAM2336	Putative (R)-3-hydroxydecanoyl-ACP: CoA transacylase, putative glycosyltransferase, putative sugar transferase	Rhamnosyltransferase chain A, Rhamnosyltransferase chain B, Rhamnosyltransferase 2	RhlA, RhlB, RhlC	-	Strong physiological evidence from Dubeau *et. *al. (2009): *Burkholderia cepacia complex *(Bcc) can synthesize rhamnolipids; high homologous similarity of RhlA, RhlB, RhlC found in *B. cenocepacia *J2315 when compared with *P. aeruginosa *PAO1 and *B. thailandensis*
BCAM2496, BCAM2497, BCAM2498	Binding-protein-dependent transport system protein, ABC transporter ATP-binding protein, extracellular solute-binding protein	Thiamin transport via ATP-binding protein	ThiP, ThiQ, ThiB	TC-3.A.1.19.1	Genetic evidence: J2315 is unable to biosynthesize thiamin, which is an important cofactor to grow, by itself and could only obtain it from culture medium; BLAST search of ThiP, ThiQ, ThiB from *E.coli *got good result
BCAM2723	Putative outer membrane porin protein	Pyroglutatmate porin OpdO	OpdO	TC-1.B.25.1.7	Evidence from BIOLOG assays; BLAST search of OpdO from *P. aeruginosa *PAO1 versus the *B. cenocepacia *J2315 genome gave an E value of 4E-32
BCAM2795	Hypothetical protein	1,4-lactonase	-	EC-3.1.1.25	BIOLOG assays indicated growth on galactose; modelling suggested a protein is missing in this pathway; BLAST search of 1,4-lactonase from *Xanthomonas campestris *gave an E value of 4E-55 and identities of 42%

The first type of refinement was to re-annotate genes in *i*KF1028 - based on literature evidence and BLAST searches - that have been improperly annotated. An example is the gene BCAL1281, which was annotated in both the *Burkholderia *Genome Database and KEGG as a "hypothetical protein", but that was now reassigned coding for an "ornithine *N*-acyltransferase". It was reported that the outer membrane of "*B. cepacia*" [62] possesses unusual polar lipids, ornithine amide lipids (OL) [63,64]. In addition, the protein OlsB is required for the first step of OL biosynthesis [65]. By BLAST searches of OlsB against the UNIPROT database, the gene BCAL1281 of *B. cenocepacia *J2315 was identified with high similarity.

Another type of annotation refinement was based on gap analyses, which pinpointed reactions for which the gene products involved were missing. For instance, we identified a missing reaction that should be catalyzed by IspE and that takes part in the biosynthesis of polyprenyl-PP, a necessary precursor of the ubiquinone biosynthesis. The IspE encoding genes for other strains of *B. cenocepacia *(AU1054, HI2424, MC0-3) could be identified in the *Burkholderia *Genome Database and KEGG. By querying GeneDB, we found that the genomic location from 872938 to 873820, named BCAL0802, was not assigned any function in the above two databases. A BLAST search of BCAL0802 against the UNIPROT database revealed a perfect match with IspE from other *B. cenocepacia *strains. Consequently, BCAL0802 is annotated as a 4-diphosphocytidyl-2-C-methyl-D-erythritol kinase and this missing reaction was supplemented into the model *i*KF1028. This example exemplifies how the reconstruction process can drive the reconciliation of isolated data from different biological databases.

BIOLOG substrate utilization assays have already been successfully used on the refinement of metabolic reconstructions [24]. It is an efficient approach for gap analysis and refinement of genome annotation. For example, in our study we can highlight the case for the substrate D-galactose, associated in BIOLOG assays with a growth phenotype. From that result we inferred that J2315 should contain a transport mechanism for D-galactose. By homology searches of MglA, MglB, and MglC, which are galactose-binding proteins conveying galactose into the cell, gene BCAL1431 (mglB), BCAL1432 (mglA), and BCAL1433 (mglC) were identified and they had been annotated as a putative sugar ABC transporter ATP-binding protein, a putative ribose ABC transport system, and a putative sugar transport system permease protein respectively. The ordering of mglB, mglA, and mglC is consistent with a) previous studies indicating that the mgl operon contains three genes and the genes are transcribed in the order of mglB, mglA, and mglC [66] and b) mglA and mglC being located downstream of mglB [67]. As a result, the annotations of BCAL1431-1433 were refined to account for the galactose transport. In total, 7 genes were reannotated based on BIOLOG assays.

### Gene essentiality analysis

**Figure 4:**
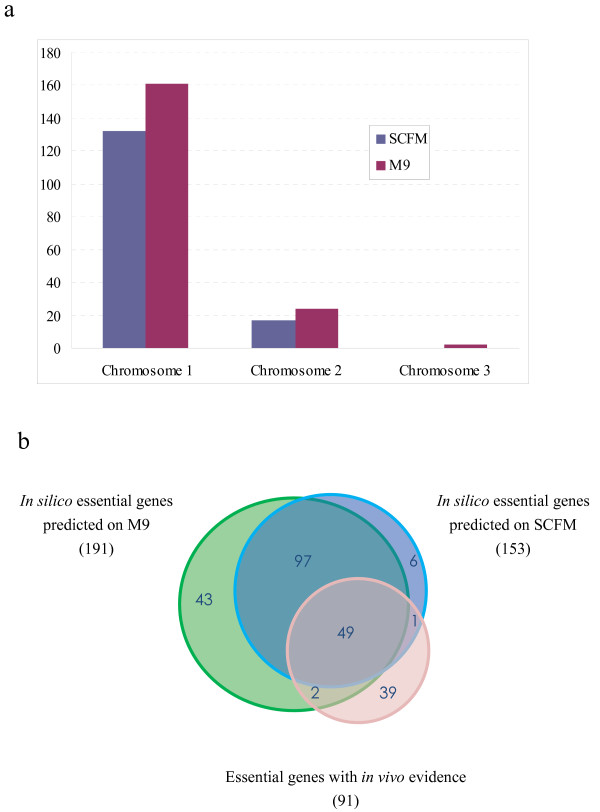
**Gene essentiality analysis**. (a) Distribution of essential genes predicted on M9 and SCFM respecively; (b) Overlapping essentail genes among *in silico *prediction on M9, SCFM, and essential genes with *in vivo *evidence from two *P. aeruginosa *strains: *P. aeruginosa *PAO1 and *P. aeruginosa *PA14.

The term 'essential gene' means a gene for which knockout is lethal (*i.e. *no biomass yield) under certain conditions (*e.g. *glucose minimal medium) [68]. Identification of essential genes is helpful to understand the basic functions required for survival and it is an efficient way to discover novel targets for new antimicrobial therapies. Here in this study, *i*KF1028 was used as a framework to predict computationally identified essential genes in *B. cenocepacia *J2315 on both M9 minimal medium and synthetic CF medium (SCFM). About 19% (192) and 15% (154) of the 1,028 metabolic genes included in *i*KF1028 were predicted to be essential on M9 and SCFM media, respectively. There are more genes predicted as essentials on M9 than on SCFM, which indicates the influence of the living environment on the bacterium. 147 overlapping predictions were on both media. These essential genes are unequally located on the three chromosomes and most of the essential genes are located on chromosome 1 (Figure 4a). This result agrees with known features of the J2315 genome: chromosome 1 contains a higher proportion of coding sequence (CDS) involved in central metabolism and other house-keeping functions, whereas chromosomes 2 and 3 contain a greater proportion of CDS encoding accessory functions [13].

To assess the predictive potential of the model, we compared the *in silico *essential genes predicted on SCFM with experimental essentiality data for *P. aeruginosa *PAO1 and *P. aeruginosa *PA14 [69,70] since there is no experimental gene essentiality data available for *B. cenocepacia*. As both *P. aeruginosa *and *B. cenocepacia *are CF pathogens, and *B. cenocepacia *was historically classified under the genus *Pseudomonas *[71], it is possible that partial similarity exists between them. SCFM was chosen due to its similarity to the nutritional composition of sputum from CF patients. The common set of the essential genes from two *P. aeruginosa *strains was chosen for comparison in order to reduce the effect of strain-dependent variation. Totally, there are 294 *in silico *essential metabolic and non-metabolic genes of *B. cenocepacia *J2315 with high similarity to the common set of *P. aeruginosa *by BLAST searches, of which 91 *in vivo *essential genes are present in *i*KF1028. Genes in the *in vivo *essential set but not in the *i*KF1028 were assumed to be involved either in non-metabolic functions or in accessory functions of metabolism. A total of 55% (50) of the *in silico *predicted essential genes agreed with the *in vivo *essential genes based on gene homology with *P. aeruginosa *(Figure 4b, refer to Additional file 4 for detailed information about essential genes).

Based on gene essentiality analysis, the genome-scale metabolic model of *B. cenocepacia *was further refined. For example, BCAL0660 and BCAL3421, which were homologous genes encoding protein AccC according to their annotation, were originally both included in the model with the gene-protein-reaction (GPR) relationship of "BCAL0660 or BCAL3421". Through *in silico *gene deletion study, BCAL3421 is identified as non-essential, which is inconsistent with the *in vivo *essential gene results. Such discrepancy was subject to further analysis. AccA, AccB, AccC, and AccD were four subunits of Acetyl-CoA Carboxylase (ACC) catalyzing the first step of fatty acid biosynthesis [72]. The gene accB, of which the locus in J2315 is BCAL3420, is frequently adjacent to the gene accC and both genes are cotranscribed and form an operon together [73,74]. Furthermore, BCAL3420 and BCAL3421 shows greater than 2-fold expression for J2315 under CF conditions versus soil conditions, yet BCAL0660 shows an opposite result [75]. Taken together, BCAL0660 was excluded from model *i*KF1028. Further studies are necessary to validate the function of BCAL0660.

### Identification of essential enzymes and potential drug targets

Essential enzyme/protein refers to a gene product (catalyzing the relevant reactions) for which individual deletion (*i.e. *imposing the fluxes through these reactions to zero) is lethal under certain conditions. Through FBA using *i*KF1028,we could obtain a collection of essential enzymes (protein), based on which 45 essential enzymes were identified as potential drug targets and supported by experimental evidences from literatures. There are 39 of them which were also predicted as drug targets for *P. aeruginosa *PAO1 [76]. All the 39 targets are nonhomologous to human protein sequences and thus could serve as potential candidate antibiotic drug targets for CF patients infected by both *B. cenocepacia *J2315 and *P. aeruginosa *PAO1. Among 39 targets, there are 9 targets, namely AccA, AccB, AccC, AccD, MurA, FolP, PhoA, RibE, and RibH, which have approved drugs in the DrugBank database [77].

The other 6 potential targets, namely ArnT, ArnB, ArnC, ArnA1, ArnA2, and Ugd are unique in *B. cenocepacia *J2315. ArnT, ArnB, ArnC, ArnA1, and ArnA2 are necessary proteins required for the synthesis of Ara4N, which is an additional moiety of LPS specially presented in *B. cenocepacia *J2315. Ara4N is essential for the viability of *B. cenocepacia *J2315 and significantly contributes to high resistance to antimicrobial peptides (AMPs) [54]. AMPs have been proposed as agents for treating CF infections [78,79]. It had also been demonstrated that arnC transposon mutant was survival-defective and attenuated in infected rats [48]. The UDP-glucose dehydrogenase (Ugd), which catalyzes the conversion of UDP-glucose to UDP-glucuronic acid and is the initial step in the synthesis of UDP-Ara4N, is also necessary for the viability of *B. cenocepacia *and its resistance to polymyxin B [80]. These targets are potentially useful for designing strategies against *B. cenocepacia *J2315. Further studies are necessary to test their applicability. An overview of the 45 proposed targets is given in Table 5.

**Table 5:** Proposed essential enzymes that can be candidate drug targets for *B. cenocepacia *J2315

Functional subsystem	EC No.	Protein	Enzyme name
Amino acid metabolism	EC-4.2.3.4	AroB	3-dehydroquinate synthase
	EC-4.2.3.5	AroC	Chorismate synthase
	EC-1.1.1.25	AroE	Shikimate dehydrogenase
	EC-1.3.1.26	DapB	Dihydrodipicolinate reductase
	EC-2.3.1.117	DapD	Tetrahydrodipicolinate succinylase
	EC-5.1.1.7	DapF	Diaminopimelate epimerase
	EC-2.7.2.4	LysC	Aspartate kinase
Lipid synthesis	EC-6.4.1.2	AccA*	Acetyl-CoA carboxylase carboxyltransferase subunit-α
	EC-6.4.1.2	AccB*	Acetyl-CoA carboxylase biotin carboxyl carrier protein subunit
	EC-6.4.1.2	AccC*	Acetyl-CoA carboxylase biotin carboxylase subunit
	EC-6.4.1.2	AccD*	Acetyl-CoA carboxylase subunit-β
	EC-2.7.8.8	PssA	Phosphatidylserine synthase
Cell wall/LPS synthesis	-	ArnA1^#^	UDP-Ara4N formyltransferase
	-	ArnA2^#^	UDP-4-keto-5-carboxypentose decarboxylase
	-	ArnB^#^	UDP-4-ketopentose aminotransferase
	-	ArnC^#^	Ara4N Und-P transferase
	-	ArnT^#^	Ara4N transferase
	EC-3.6.1.27	BacA	Undecaprenyl pyrophosphate phosphatase
	EC-2.5.1.55	KdsA	2-dehydro-3-deoxyphosphooctonate aldolase
	EC-2.7.7.38	KdsB	3-deoxy-manno-octulosonate cytidylyltransferase
	EC-2.3.1.129	LpxA	UDP-N-acetylglucosamine acyltransferase
	EC-2.4.1.182	LpxB	Lipid-A-disaccharide synthase
	EC-3.5.1.-	LpxC	UDP-3-O-[3-hydroxymyristoyl]N-acetylglucosamine deacetylase
	EC-2.7.1.130	LpxK	Tetraacyldisaccharide 4'-kinase
	EC-2.5.1.7	MurA*	UDP-N-acetylglucosamine 1-carboxyvinyltransferase
	EC-1.1.1.158	MurB	UDP-N-acetylmuramate dehydrogenase
	EC-6.3.2.8	MurC	UDP-N-acetylmuramate--L-alanine ligase
	EC-6.3.2.9	MurD	UDP-N-acetylmuramoyl-L-alanyl-D-glutamate synthetase
	EC-6.3.2.13	MurE	UDP-N-acetylmuramoylalanyl-D-glutamate--2, 6-diaminopimelate ligase
	EC-2.4.1.227	MurG	Undecaprenyldiphospho-muramoylpentapeptide-β-N-acetylglucosaminyltransferase
	EC-1.1.1.22	Udg^#^	UDP-glucose dehydrogenase
	EC-2.4.1.-	WaaF	UDP-glucose:(heptosyl) LPS-α-1,3-glucosyltransferase
	EC-5.1.3.13	RmlC	dTDP-4-dehydrorhamnose 3,5-epimerase
Vitamin and cofactor synthesis	EC-2.7.11.5	AceK	Bifunctional isocitrate dehydrogenase kinase/ phosphatase protein
	EC-4.1.2.25	FolB	Dihydroneopterin aldolase
	EC-2.5.1.15	FolP*	Dihydropteroate synthase
	EC-1.2.1.70	HemA	Glutamyl-tRNA reductase
	EC-2.1.2.11	PanB	3-methyl-2-oxobutanoate hydroxymethyltransferase
	EC-6.3.2.1	PanC	Pantoate--β-alanine ligase
	EC-1.1.1.169	PanE	2-dehydropantoate 2-reductase
	EC-3.1.3.1	PhoA*	Alkaline phophatase
	EC-3.5.4.25	RibB	Bifunctional 3,4-dihydroxy-2-butanone 4-phosphate synthase
	EC-3.5.4.26	RibD	Riboflavin-specific deaminase/reductase
	EC-2.5.1.9	RibE*	Riboflavin synthase subunit-α
	EC-2.5.1.9	RibH*	6,7-dimethyl-8-ribityllumazine synase

^# ^Essential enzymes unique for *B. cenocepacia *J2315 (others are shared between *B. cenocepacia *J2315 and *P. aeruginosa *PAO1).

* The proteins have had approved drugs from the DrugBank database.

## Conclusions

In this study, we reconstructed the first manually curated genome-scale metabolic network of *B. cenocepacia *J2315, a Gram-negative pathogen for CF patients. An iterative reconstruction process led to the establishment of the model, termed *i*KF1028, which captures the important metabolic capabilities and biosynthesis of key metabolic virulence factors. The model *i*KF1028 shows its predictive potential when compared with BIOLOG assays. Model-driven analyses on gene annotation refinement and identification of gene and enzyme essentiality analyses are helpful to understand the genome and discover promising novel drug targets. Through careful investigation, we proposed 45 enzymes that catalyze reactions predicted to be essential for growth with priority to be considered as drug targets. The model will keep being further validated and improved with experimentally determined biomass composition, large-scale gene deletion experimental data, proteome, and metabolome data, as they become available for *B. cenocepacia*. The model herein developed provides a valuable tool to explore the metabolic space of *B. cenocepacia*, to describe its metabolic wiring under a range of conditions, to pinpoint possible targets and to generate testable hypotheses. Taken together, our study underlined the value of the model *i*KF1028 as a framework to systematically study the metabolic capabilities of *B. cenocepacia *and its metabolic virulence factors affecting the CF community.

## Methods

### Reconstruction of the metabolic network

**Figure 5:**
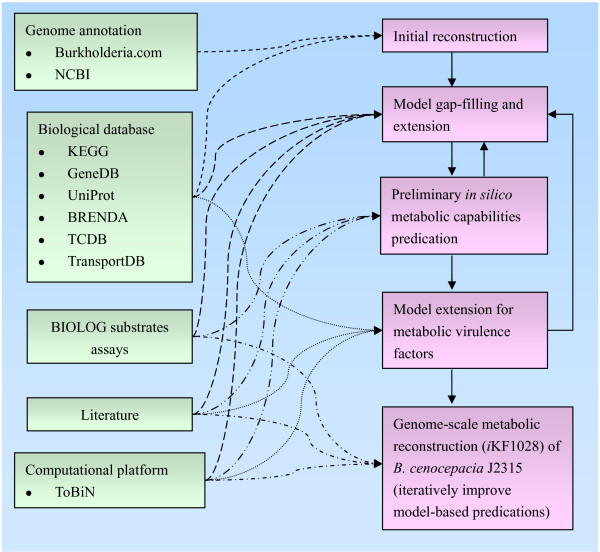
**The process for genome-scale metabolic reconstruction of *B. cenocepacia *J2315**. The left side indicates resources used for reconstruction, and the right side indicates the reconstruction process. Initial reconstruction started from genome annotation and other biological databases. Gap-filling was a continuous step throughout the reconstruction by probing missing reactions in a pathway which causes *in silico *growth infeasible, and subsequently closing these gaps by referring to the biological databases, extensive literature mining, and comparison with BIOLOG substrate utilization assays [89,90]. This improved model was then extended by adding key metabolic virulence factors for *B. cenocepacia *from the literature. The process of model development and validation against experimental data was iteratively repeated until the genome-scale metabolic model was robust.

The reconstruction process for *B. cenocepacia *J2315 is illustrated in Figure 5. The process followed the procedure described previously [81]. The reconstruction was carried out on ToBiN (Toolbox for Biochemical Networks, http://www.lifewizz.com), which was first mentioned in the paper [82]. ToBiN is a modular platform for metabolic modelling and the structural analysis of networks. It consists of a collection of open-source computational tools. Sets of reactions can be uploaded in the platform via a web interface, merged with already existing sets, and the resulting stoichiometric matrix is then processed by the server as a FBA problem. The linear solver that ToBiN used is the Clp (Coin-or linear programming), an open-source linear programming solver written in C++ and is part of the COIN-OR (Computational Infrastructure for Operations Research) project (http://www.coin-or.org). The platform works in a similar way as the COBRA toolbox with the main difference that, by being web-based, it permits users to adopt a more efficient and collaborative workflow.

An initial draft reconstruction was derived from the annotated genome of *Burkholderia cenocepacia *J2315 available at the *Burkholderia *Genome Database (http://www.burkholderia.com). To link annotated genes to proteins and proteins to reactions, biological databases such as KEGG, GeneDB, UniProt, BRENDA, Transport Classification Database (TCDB), and TransportDB were used [83-88]. Manual curations were performed to establish gene-protein-reaction (GPR) associations, which connect genetic data to reactions in the metabolic network and allow for subsequent exploration of metabolic phenotypes using genetic perturbations.

After the initial reconstruction was generated, gaps in metabolic pathways necessary to produce biomass components and key virulence factors were filled by cautious literature mining, BIOLOG substrates utilization assays, and BLAST searches on homology and protein sequence similarity analyses [89,90]. The genome annotation was refined as consequence of the gap-filling and model extension process.

Flux Balance Analysis (FBA) was carried out throughout this study to explore the metabolic capabilities of *i*KF1028 under various environments. In addition to minimal medium, synthetic cystic fibrosis medium (SCFM) representing the physical living environment during CF infection was simulated *in silico *to investigate the metabolic flux distribution in a CF-like condition.

### Biomass composition

The biomass composition in the genome-scale metabolic model of *B. cenocepacia *J2315 was adapted by selecting the well-studied biomass composition of *E. coli *as a template [91], since there's no experimental data available about the biomass composition of *B. cenocepacia*. However, the amount of metabolic precursors to formulate the cellular component was specific to *B. cenocepacia *according to previous study [20]. Moreover, the relative fatty acid composition of the lipids required for growth was based on data specific to *B. cenocepacia *[63,64,92,93] and listed in Table 6. Further details are provided in the supplemental material [Additional file 5].

**Table 6:** Amino lipid composition of *B. cenocepacia *J2315

Fatty acid		PE	PG	CLPN	OL
Saturated	16:0	+	+	+	ND
Unsaturated	16:1	+	+	+	ND
	18:1	+	+	+	+
Hydoxy	14:0 3OH	ND	ND	ND	+
	16:0 3OH	ND	ND	ND	+
Cyclopropane	17:0 CYC	+	+	+	ND
	19:0 CYC	+	+	+	ND

PE, phosphatidylethanolamine; PG, phosphatidylglycerol; CLPN, cardiolipin; OL, ornithine amide lipid; ND, not determined; plus symbol (+), fatty acid was detected in a significant amount

### Flux balance analysis

Flux balance analysis (FBA) is an algorithm based on linear programming (LP) and on the assumption that the represented metabolic network is in steady-state (i.e. all the intracellular metabolite concentrations are constant). Being a LP problem, FBA also requires the selection of an objective function and of whether the value for that same function should be maximized or minimized. FBA is usually used to compute the optimal growth yield (the maximized objective function) based on the assumption that the evolutionary fitness of the organism depends on growth alone and, consequently, the implicit regulatory mechanism are organized to permit the theoretical maximal growth. If the system of equations (stoichiometric matrix which represents the metabolic network) is feasible, the algorithm generates an optimal flux distribution for that same network, taking into account the imposed thermodynamic constraints (reaction directionality) and limits on substrate uptake rates. The mathematical description is as follows:

Where *S *is a stoichiometric matrix containing *i *rows representing metabolites and *j *columns representing reactions, *v *is a vector of all reaction fluxes, *v*_min _and *v*_max _are imposed lower and upper bounds on flux *v*_*j *_respectively, and *c*^*T *^is a vector of coefficients for each reaction that is to be maximized.

### *In silico *media composition

Two different living environments were simulated *in silico *for strain J2315: M9 minimal medium [94], which contains PO_4_^3-^, SO_4_^2-^, NH_4_^+^, H^+^, Fe^2+^, K^+^, Mg^2+^, Na^+^, H_2_O, and thiamine, with glucose or other BIOLOG substrates as sole carbon source; and synthetic CF sputum medium (SCFM) [95] representing the nutrient conditions inside a host-cell during CF infection. Details of the simulated SCFM composition are provided in the supplemental material [Additional file 6].

### BIOLOG assay

To validate the model and estimate the metabolic capabilities of strain J2315, BIOLOG assay was performed by using various carbon sources for strain cultivation [16]. The BIOLOG assay was carried out in triplicates using Biolog GN2 MicroPlates (Biolog, Inc.), which can test the ability of a microorganism to oxidize a panel of 95 different carbon sources simultaneously. The procedure for using the MicroPlates was according to the manufacturer's specification. The strain J2315 was obtained from DSMZ GmbH (DSMZ 16553, equivalent to LMG 16656 as which strain J2315 has been deposited in the BCCM/LMG Bacteria Collection). The strain was cultured overnight in CASO agar plate. Then the bacteria were swabbed from the plate surface and suspended in GN/GP inoculating fluid (Biolog, Inc.) and 150 μl of the suspension was transferred to each well of the GN2 MicroPlate. The MicroPlates were incubated at 30°C for 48 hours and were read by a microplate reader at 24 and 48 hours and analyzed with the Biolog MicroLog3 4.20 software (Biolog, Inc.). A comparison between the BIOLOG results and *in silico *predictions is provided in the supplemental material. [Additional file 3]

### Gene and enzyme essentiality

FBA can be used to interpret genetic modification, such as gene deletion and enzyme inhibition, and subsequently make comprehensive *in silico *predictions on gene and enzyme essentiality [96]. To assess the essentiality of a gene, its GPR is checked for a unique relation with the associated reaction(s). If the gene is necessary to the reaction, the reaction flux will be constrained to zero and a solution for the maximal growth yield is searched. The deleted gene is predicated to be essential if, as consequence of that added constraint, the value of the objective function (growth yield) changes to zero. The deletion of every gene accounted in the model was simulated for growth on minimal medium with glucose as sole carbon source, and on SCFM. Similarly, an enzyme is considered essential if, by constraining to zero the flux on every associated reaction that has no alternative means of catalysis, the value for the growth yield changes to zero. The essentiality of every enzyme accounted in the model was analysed for growth on SCFM.

## Authors' contributions

KCF, HSZ, SCY, SHC, and KLZ carried out the reconstruction of *Burkholderia cenocepacia *J2315. KCF designed the study and performed the analysis. KCF and CMCL arranged the BIOLOG substrate utilization analysis. JW and VMDS supervised the research. KCF, CMCL, VMDS, and JW drafted the manuscript. GP provided technical support for the reconstruction of *B. cenocepacia *J2315 in ToBiN. MG was involved in the early stage of the development of *i*KF1028 in ToBiN. All authors read and approved the final manuscript.

## Supplementary Material

Additional file 1 Reconstructed metabolic network model *i*KF1028 Click here for file

Additional file 2 Model *i*KF1028 in SBML format Click here for file

Additional file 3 BIOLOG assays Click here for file

Additional file 4 Gene essentiality comparison results Click here for file

Additional file 5 Biomass detailed information Click here for file

Additional file 6 Synthetic CF medium simulated *in silico* Click here for file
